# The Occurrence of Single-Site Pharyngeal *Neisseria gonorrhoeae* Among Female Sex Workers in the Netherlands

**DOI:** 10.1097/OLQ.0000000000002104

**Published:** 2024-11-21

**Authors:** Ymke J. Evers, Charlotte M.M. Peters, Petra F.G. Wolffs, Dieuwertje L. Horsten, Chantal Weijzen, Nicole H.T.M. Dukers-Muijrers, Christian J.P.A. Hoebe

**Affiliations:** From the ∗ Department of Sexual Health, Infectious Diseases and Environmental Health, Living Lab Public Health MOSA, South Limburg Public Health Service, Heerlen; † Department of Social Medicine, Care and Public Health Research Institute (CAPHRI), Maastricht University; ‡ Department of Medical Microbiology, Infectious Diseases & Infection Prevention, National *Chlamydia trachomatis* Reference Laboratory, Care and Public Health Research Institute (CAPHRI), Maastricht University Medical Center (MUMC+); § Department of Health Promotion, Care and Public Health Research Institute (CAPHRI), Maastricht University, Maastricht, the Netherlands

## Abstract

**Background:**

Female sex workers (FSWs) have a higher burden of sexually transmitted infections (STI), including *Neisseria gonorrhoeae* (NG), than the non–sex worker population, mainly due to social vulnerability and work-related factors. This large study evaluated the anatomical site distribution of NG, and specifically the occurrence of single-site pharyngeal NG, among FSWs visiting Dutch STI clinics in the Netherlands.

**Methods:**

Coded STI clinic consultations from FSW attending any Dutch STI clinic between 2016 and 2021, in which tests were performed on all 3 anatomical sites, according to the Dutch testing policy for FSW, were included in analyses (n = 22,304). To compare the anatomical site distribution of NG among FSWs with women in general, 15,494 consultations from women who were tested on 3 anatomical sites in clinics that performed standard testing at 3 anatomical sites were used. Descriptive statistics were used to compare the anatomical site distribution of NG and occurrence of single-site pharyngeal NG among FSW and universally tested women.

**Results:**

NG was diagnosed among 3.5% (782 of 22,034) among FSWs and 1.7% (271 of 15,494) among universally tested women (*P* < 0.001). Pharyngeal NG was diagnosed among 2.3% of FSWs versus 0.8% of universally tested women (*P* < 0.001). Single-site pharyngeal NG was diagnosed in 19.9% (156 of 782) of all NG infections, compared with 14.8% (40 of 271) of NG infections among universally tested women.

**Conclusion:**

The substantial proportion pharyngeal NG and single-site pharyngeal NG among FSW confirms the relevance of pharyngeal testing among this key population for optimal patient management, also in the context of the potential role of pharyngeal NG in antimicrobial resistance.

## Abstract 2

A higher positivity rate of *Neisseria gonorrhoeae* (NG) and both concurrent and single-site pharyngeal NG was assessed among female sex workers than in universally tested women visiting Dutch sexually transmitted infection clinics.

Gonorrhea is a sexually transmitted infection (STI) caused by the bacterium *Neisseria gonorrhoeae* (NG), which can infect genital, anorectal, or pharyngeal mucosa with subsequent symptoms. When left untreated, symptoms might persist and NG infections might lead to complications such as cervicitis, pelvic inflammatory disease, ectopic pregnancy, and infertility in women.^1^ Reports suggested increasing incidences of NG infections in several countries in Europe and North America.^2,3^ The threat of emerging antibiotic resistance of NG against the first-line treatment, ceftriaxone, is concerning. The World Health Organization has therefore classified NG as a priority pathogen and declared it an urgent global public health concern.^4^ There is no highly effective vaccine, making effective testing strategies and antimicrobial treatment in key populations crucial to control NG.^5^ One of these key populations includes female sex workers (FSWs), as they are at high risk for NG acquisition and transmission, due to their structural vulnerability and factors related to their work such as multiple sex partners, inconsistent condom use during oral sex, or previous STI diagnoses.^6–8^

A systematic review in Europe showed a prevalence of 3.2% (95% confidence interval, 1.8%–4.8%) genital NG among FSWs, compared with 1.0% (95% confidence interval, 0.7%–1.2%) in the general population.^9^ Based on national surveillance in the Netherlands, a positivity rate of 4.7% of NG has been assessed among FSWs visiting the Public Health Service STI clinics in 2022.^10^ Previous research has assumed that the pharynx might play an important role in gonococcal transmission,^11^ possibly due to the estimated duration of untreated pharyngeal NG being 16 weeks.^12^ Moreover, the pharynx probably plays a critical role in the development of antimicrobial resistance (AMR).^13,14^ A previous Dutch study among women visiting STI clinics showed that sex work was an independent risk factor for pharyngeal NG.^15^ Extragenital NG testing policy is mostly advocated on indication by the health care professional based on sexual history (e.g., having genital sex with condom and condomless pharyngeal sex) and in several countries (e.g., in the Netherlands) indicated in key populations in which burden is higher, such as sex workers.^16–18^ Most countries have not included sex work as an indication for pharyngeal NG testing, which might result in missed infections. There is a knowledge gap on the occurrence of single-site pharyngeal NG and concurrency with other anatomic sites. Such knowledge helps to understand whether universal testing of all FSW at both genital and pharyngeal sites would be warranted. Therefore, the current study evaluates the anatomical site distribution, including the occurrence of single-site pharyngeal NG, among FSWs visiting Dutch STI clinics. As a frame of reference, we compared the anatomical site distribution of NG among FSWs with women who visited STI clinics that standardly tested all women on 3 anatomical sites.

## METHODS

### Study Design

In this retrospective cohort study, coded surveillance consultations of FSW were included from all outpatient Public Health STI clinics (also known as Sexual Health Centers) in the Netherlands (25 Public Health Services with 38 STI clinic locations), which were submitted between January 1, 2016, and December 31, 2021, via an electronic patient registry using a consultation code, to the National Institute of Public Health and the Environment. Reporting of data to this national institute is standardized and mandatory for all STI clinics, thereby covering virtually all consultations performed. The publicly funded Dutch STI clinics serve high-risk groups, including sex workers, young people younger than 25 years, and men who have sex with men. Sex workers can visit these clinics for free testing and sexual health counseling. Some STI clinics also perform outreach activities for sex workers. Sex work was defined as having sex in exchange for money or goods. In the national testing policy, sex work includes an indication for universal testing, which means testing on all 3 anatomical sites for CT and NG (genital, anorectal, and pharyngeal site).^19^

Although it is not the general Dutch policy to test all women for NG on 3 anatomical sites, several STI clinics performed universal testing. Therefore, we were able to create a reference group consisting of women who were universally tested on 3 anatomical sites in the dataset, independent of specific behavior or symptoms and thereby minimizing selection bias. This classification of whether a clinic performed routine universal testing was based on practical experience used in previous research.^15,20^ A clinic calendar year was defined as routine universal testing when at least 80% of women attending the clinic that year were tested on 3 anatomical sites. Although routine universal testing implies testing on 3 anatomical sites in all women, achieving 100% coverage has been proven unfeasible in practice, for example, in case of opting out for anorectal or pharyngeal testing by clients. In this study, we included data of consultations from FSW and women who visited STI clinics that performed routine universal testing, and when they were tested on all 3 anatomical sites (see flowchart Supplementary File I, http://links.lww.com/OLQ/B158).

Of both groups, we extracted consultation-level data and individual-level data from 2016 onward for sociodemographic characteristics and sexual behavior (e.g., sex work) in the past 6 months from patient registry forms verified by STI nurses and STI diagnoses.

### Statistical Analysis

We described the study population and compared the FSW and universally tested women by sociodemographic characteristics and STI positivity rates. NG positivity rates were calculated by dividing the number of positive NAAT test results by the total number of tests. Within all NG positive test results, the proportion of single-site NG infections was calculated as the number of positive test results on one site without any concurrent NG infections. The proportion of single and concurrent infections was shown in a Venn diagram (created in www.metachart.com). The presented Venn diagram is based on all included consultations of our study population. We also created Venn diagrams for all first consultations in our database on individual level (including only one consultation for each unique person). This Venn diagram was very similar and presented in Supplementary File II, http://links.lww.com/OLQ/B158.

## RESULTS

### Study Population

The median age of FSW was 31 years (interquartile range, 27–36), and the median age of universally tested women was 22 years (interquartile range, 21–24). The majority, both FSW and universally tested women, had a Western ethnicity. A large proportion of educational level among FSWs was unknown, mostly due to shortened registration in venues. Any STI was diagnosed among 10.5% (2346 of 22,304) of FSW and 17.3% (2679 of 15,494) of universally tested women (*P* < 0.001; Table 1).

**TABLE 1:** Characteristics and STI Diagnoses Among FSWs and Routinely Tested Women (Description of Study Population)

			
	FSW, Tested on 3 Anatomical Sites	Routinely Tested Women, Tested on 3 Anatomical Sites	*P* Value*χ*^2^/Fisher exact
On a person level	N = 7857	N = 11,552	
Age, y			<0.001
≤21	9.9 (774)	51.0 (5886)	
22–28	29.1 (2289)	36.0 (4163)	
≥29	61.0 (4794)	13.0 (1503)	
Ethnicity			<0.001
Western	67.9 (5335)	73.1 (8439)	
Non-Western	31.6 (2480)	26.8 (3095)	
Unknown	0.5 (42)	0.2 (18)	
Education*			<0.001
High	19.1 (1499)	56.9 (6578)	
Medium	10.8 (846)	15.7 (1818)	
Low	30.6 (2404)	24.0 (2776)	
Other/unknown	39.5 (3108)	3.3 (380)	
On a consultation level	N = 22,304	N = 15,494	
Any STI^†^	10.5 (2346)	17.3 (2679)	<0.001
*C. trachomatis*	7.5 (1664)	16.3 (2521)	<0.001
New HIV	0.1 (12)	0.0 (6)	0.634
Infectious syphilis	0.1 (24)	0.0 (7)	<0.001
Acute hepatitis B	0.1 (32)	0.0 (6)	<0.001
*N. gonorrhoeae*^‡^	3.5 (782)	1.7 (271)	<0.001
Genital NG	2.6 (580)	1.3 (207)	<0.001
Anorectal NG	1.5 (332)	1.2 (181)	0.008
Pharyngeal NG	2.3 (523)	0.8 (131)	<0.001
On a person level			
*N. gonorrhoeae*	3.9 (308)	1.7 (194)	<0.001
Genital NG	2.9 (231)	1.3 (149)	<0.001
Anorectal NG	1.7 (134)	1.2 (133)	0.001
Pharyngeal NG	2.5 (193)	0.7 (86)	<0.001
Of all consultations with NG diagnosis	N = 782	N = 271	
Genital-single	12.0 (94)	11.4 (31)	0.798
Anorectal-single	3.7 (29)	8.9 (24)	0.002
Pharyngeal-single	19.9 (156)	14.8 (40)	0.054
Genital-anorectal	17.4 (136)	31.4 (85)	<0.001
Genital-pharyngeal	25.6 (200)	7.0 (19)	<0.001
Anorectal-pharyngeal	2.2 (17)	0.0 (0)	0.010
Genital-anorectal-pharyngeal	19.2 (150)	26.6 (72)	0.012

*Level of education was categorized into practical: elementary, prevocational secondary; mixed: senior general secondary, pre-university, secondary vocational; and theoretical: higher professional, university based on the definitions used by Statistics Netherlands (www.cbs.nl).

^†^Any STI was defined as being diagnosed with *Chlamydia trachomatis*, *Neisseria gonorrhoeae*, new HIV infection, infectious syphilis, or acute hepatitis B.

^‡^NG diagnosis is based on a positive NAAT test result. All involved medical microbiological laboratories use commercially available NAATs, which are FDA-approved tests according to the manufacturer's protocol, and in all involved laboratories, qualitative assessment and proficiency testing is in place.

### Positivity Rates of NG at Different Anatomic Sites

**Figure 1:**
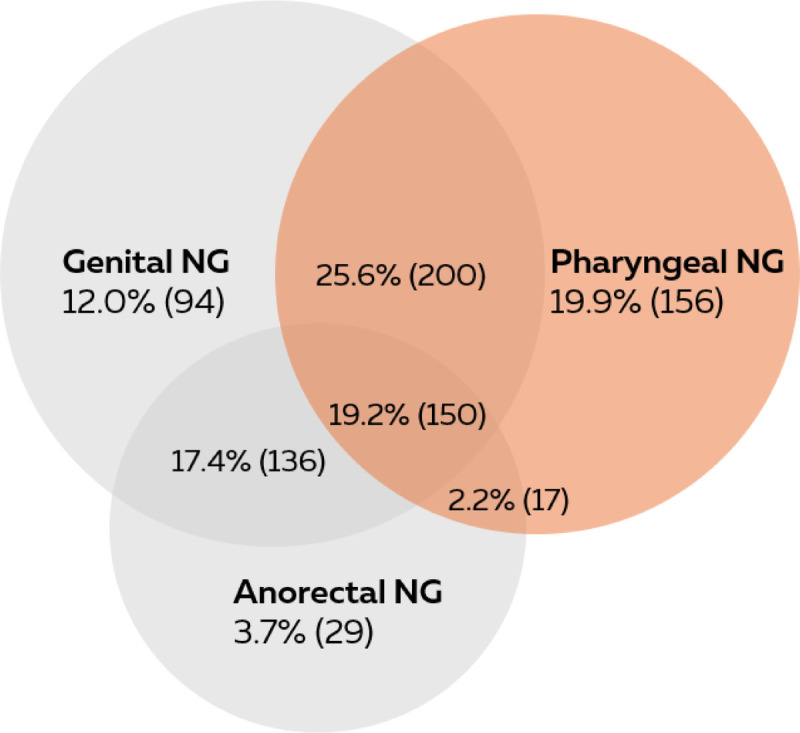
Venn diagram of the anatomical site distribution of *N. gonorrhoeae* in FSW (n = 782 consultations with NG diagnosis) visiting Dutch STI clinics (2016–2021), presented on consultation level.

NG was diagnosed among 3.5% (782 of 22,304) of FSWs and 1.7% (271 of 15,494) of routinely tested women (*P* < 0.001). Pharyngeal NG (2.3%) was significantly more occurrent among FSWs than among universally tested women (0.8%, *P* < 0.001), and single-site pharyngeal NG was diagnosed in 19.9% of all NG infections (Fig. 1). Both single-site pharyngeal NG and concurrent pharyngeal-genital and pharyngeal-anorectal infections were more common among FSWs than among universally tested women (Table 1).

## DISCUSSION

This large study provides a comprehensive overview of the anatomical site distribution of NG, focused on pharyngeal NG, among FSWs, and we compared this distribution to universally tested women on 3 anatomical sites. Overall, NG positivity in FSW was higher than in universally tested women. Pharyngeal NG was also higher among FSWs than among universally tested women, consisting of both more single-site and concurrent infections. The high proportion of single-site pharyngeal NG underpins the relevance of pharyngeal NG testing in addition to genital testing among FSWs.

The anatomical site distribution of NG infections is scientifically and societally relevant, as pharyngeal infections are recently suggested to be of importance in the emergence and transmission of AMR. Previous research has already shown that extragenital NG infections are often missed in women, as testing policies generally do not indicate extragenital NG testing in women.^15^ Without pharyngeal testing, 1 in 5 NG infections would be missed in FSWs. Incorporation of sex work as indication for pharyngeal NG testing in testing policies and following adequate detection and treatment of these pharyngeal infections might optimize patient and AMR management. It is advisable to include a question on receiving money or goods in exchange for sex and offer self-sampled pharyngeal testing. Future research is needed in the role of pharyngeal NG in AMR and the benefits of asymptomatic NG screening. For *Chlamydia trachomatis,* there is currently a shift in testing less asymptomatic persons (syndromic management), as it less often leads to serious health burden and pharyngeal infections are scarce and mostly self-limiting.^21^ To date, the state of the art is that NG testing remains a crucial part of STI control, as NG has a higher potential for causing severe complications and its propensity for developing AMR.

We used universally tested women as a reference group to compare our findings in sex workers with. We created this reference group based on clinic years in which at least 80% of women were standard tested at 3 anatomical sites, which eliminated selection bias based on indications for extragenital testing in this group. However, this reference group reflects the Dutch STI clinic testing policy to test many young people younger than 25 years and therefore deviate in sociodemographic characteristics and, for example, CT positivity rates from FSWs. Although we do not anticipate that sociodemographic factors, that is, age and ethnicity, determine anatomical site distribution, it is essential to interpret our study results with this limitation in mind. Furthermore, FSWs had more repeat consultations than universally tested women, and this could potentially have impacted the results. However, we analyzed the data also on a person level, and this resulted in very similar results. In another sensitivity analysis, we excluded consultations within 1-month time interval to minimize the risk of counting same NG infections, but this resulted in identical findings. This indicates robustness of our finding that pharyngeal NG occurrence is higher among FSWs than universally tested women.

In conclusion, our findings highlight the importance of including pharyngeal NG testing alongside genital testing to better address and manage the higher infection rates observed in FSWs compared with universally tested women.
